# Standard versus personalized schedule of regorafenib in metastatic gastrointestinal stromal tumors: a retrospective, multicenter, real-world study

**DOI:** 10.1016/j.esmoop.2021.100222

**Published:** 2021-08-02

**Authors:** M. Nannini, A. Rizzo, M.C. Nigro, B. Vincenzi, A. Mazzocca, G. Grignani, A. Merlini, L. D’Ambrosio, F. Tolomeo, G. Badalamenti, L. Incorvaia, A. Bonasera, E. Fumagalli, D. Miliziano, F. Ligorio, A. Brunello, B. Chiusole, S. Gasperoni, M. Novelli, M.A. Pantaleo

**Affiliations:** 1 Division of Oncology, IRCCS Azienda Ospedaliero-Universitaria di Bologna, Bologna, Italy; 2 Department of Oncology, University Campus Bio-Medico, Rome, Italy; 3 Division of Medical Oncology, Candiolo Cancer Institute, FPO – IRCCS, Candiolo, Italy; 4 Section of Medical Oncology, Department of Surgical, Oncological and Oral Sciences, University of Palermo, Palermo, Italy; 5 Adult Mesenchymal Tumour and Rare Cancer Medical Oncology Unit, Fondazione IRCCS Istituto Nazionale dei Tumori, Milan, Italy; 6 Medical Oncology 1, Department of Oncology, Istituto Oncologico Veneto IOV - IRCCS, Padova, Italy; 7 Translational Oncology Unit, Azienda Ospedaliero-Universitaria Careggi, Firenze, Italy; 8 Department of Statistical Sciences, University of Bologna, Bologna, Italy; 9 Department of Experimental, Diagnostic and Specialty Medicine, Sant’Orsola-Malpighi University Hospital, Bologna, Italy

**Keywords:** GIST, regorafenib, personalized treatment, toxicity, quality of life

## Abstract

**Background:**

Despite its proven activity as third-line treatment in gastrointestinal stromal tumors (GIST), regorafenib can present a poor tolerability profile which often leads to treatment modifications and transient or permanent discontinuation; thus, in clinical practice physicians usually adopt various dosing and interval schedules to counteract regorafenib-related adverse events and avoid treatment interruption. The aim of this real-world study was to investigate the efficacy and safety of personalized schedules of regorafenib in patients with metastatic GIST, in comparison with the standard schedule (160 mg daily, 3-weeks-on, 1-week-off).

**Patients and methods:**

Institutional registries across seven Italian reference centers were retrospectively reviewed and data of interest retrieved to identify patients with GIST who had received regorafenib from February 2013 to January 2021. The Kaplan–Meier method was used to estimate survival and the log-rank test to make comparisons.

**Results:**

Of a total of 152 patients with GIST, 49 were treated with standard dose, while 103 received personalized schedules. At a median follow-up of 36.5 months, median progression-free survival was 5.6 months [95% confidence interval (CI) 3.73-11.0 months] versus 9.7 months (95% CI 7.9-14.5 months) in the standard-dose and the personalized schedule groups, respectively [hazard ratio (HR) 0.51; 95% CI 0.34-0.75; *P* = 0.00052]. Median overall survival was 16.6 months (95% CI 14.1-21.8 months) versus 20.5 months (95% CI 15.0-25.4 months), respectively (HR 0.75; 95% CI 0.49-1.22; *P* = 0.16).

**Conclusions:**

Regorafenib-personalized schedules are commonly adopted in daily clinical practice of high-volume GIST expert centers and correlate with significant improvement of therapeutic outcomes. Therefore, regorafenib treatment optimization in patients with GIST may represent the best strategy to maximize long-term therapy.

## Highlights

• Regorafenib-personalized schedules are commonly adopted in daily clinical practice.
• Regorafenib-personalized schedules correlate with statistically significant improvement of therapeutic outcomes.
• A prompt personalization of regorafenib could help clinicians avoid early treatment discontinuation due to adverse events.
• A patient-tailored approach could be applied to other metastatic solid tumors treated with regorafenib.

## Introduction

The last 20 years have witnessed important advances in the medical management of gastrointestinal stromal tumor (GIST), the most commonly diagnosed mesenchymal neoplasm of the digestive tract.^1^^,^^2^ In fact, although no effective therapeutic options were available until the early 2000s, the identification of the role of Proto-Oncogene Tyrosine-Protein Kinase Kit (KIT) and platelet-derived growth factor receptor A (PDGFRA) in the oncogenesis of these malignancies has led to the approval of tyrosine-kinase inhibitors, including imatinib, sunitinib, regorafenib, and ripretinib and avapritinib.^3^^,^^4^

Regorafenib is an oral multikinase inhibitor that targets several protein kinases, including those implied in the regulation of proliferation (KIT, RET, RAF-1, BRAF, and BRAF V600E), angiogenesis [vascular endothelial growth factor receptor-1 (VEGFR1), VEGFR2, VEGFR3, and tie-like receptor tyrosine kinase 3 (TIE3)], and tumor microenvironment [PDGFR and fibroblast growth factor receptor (FGFR)].^5^ In recent years, regorafenib has reported antitumor activity in several phase I to III clinical trials evaluating this molecule in different settings, including heavily pretreated metastatic colorectal cancer, hepatocellular carcinoma, and metastatic GIST.^6^, ^7^, ^8^ Moreover, regorafenib is currently being investigated as a monotherapy or in combination with other anticancer agents in several other malignancies, and thus, the number of indications of regorafenib is supposed to further increase in the near future.^9^, ^10^, ^11^ Concerning GIST, regorafenib significantly improved progression-free survival (PFS) in patients with advanced disease progressing after failure of at least imatinib and sunitinib in the phase III GRID trial.^11^ In this study, median PFS was 4.8 months in patients receiving regorafenib, compared with 0.9 months in the placebo group [hazard ratio (HR) 0.27; 95% confidence interval (CI) 0.19-0.39; *P* < 0.0001]. In addition, disease control rate was 52.5% (70/133) and 9.1% (6/66) in the regorafenib and the placebo group, respectively. Finally, no statistically significant difference in overall survival (OS) was observed, because cross-over was allowed (HR 0.77, 95% CI 0.42-1.41; *P* = 0.199).^12^

The standard recommended dose of regorafenib is 160 mg/day, once daily, for 3 weeks followed by 1 week off therapy.^13^ Despite its undoubted efficacy, regorafenib has been associated with several grade 1-4 adverse events (AEs), including hand-foot skin reaction, rash, stomatitis, diarrhea, hypertension, and fatigue.^14^^,^^15^ Of note, these drug-associated AEs often require dose adaptation and transient—or even definitive—treatment interruption.^16^ Thus the use of regorafenib is challenging in the real-life setting, and recent years have seen the adoption of various dosing or interval schedules by clinicians worldwide, to improve patient adherence. Nevertheless, there is a surprising overall paucity of data.^17^^,^^18^ Moreover, no data on the impact of personalized schedules of regorafenib on therapeutic outcomes in patients with advanced GIST have been collected.

On these premises, we aimed to investigate the efficacy and safety of both personalized and standard schedules of regorafenib in patients with metastatic GIST, in the real-life Italian clinical setting.

## Patients and methods

### Patients

A retrospective multicenter study including all patients diagnosed with recurrent, locally advanced, or metastatic GIST treated with regorafenib at seven Italian reference centers (Bologna GIST Study Group – IRCCS Azienda Ospedaliero-Universitaria Bologna, Bologna; Istituto Nazionale Tumori, Milan; University Campus Bio-Medico, Rome; Policlinico “Paolo Giaccone”, Palermo; Candiolo Cancer Institute, Candiolo; Istituto Oncologico Veneto IOV, Padova; Azienda Ospedaliero-Universitaria Careggi, Firenze) was performed between February 2013 and January 2021. Major eligibility criteria for inclusion of patients were histologically proven GIST, unresectable locally advanced or metastatic disease, age ≥18 years, and receipt of at least one cycle of regorafenib. Patients treated with regorafenib were split into two groups: (1) patients with GIST treated with regorafenib at the standard schedule (schedule of 160 mg once daily for the first 3 weeks of each 4-week cycle) for the entire duration of regorafenib treatment; (2) patients with GIST that received personalized schedules of regorafenib either upfront or after treatment adjustment due to intolerance.

Baseline clinicopathologic and laboratory data were retrieved from the institutional registries of the participating centers through electronic medical records review. For each patient, the following variables were collected and analyzed: age, primary tumor site, risk class, mutational status, disease status at diagnosis, adjuvant treatment, and number of prior lines of therapy.

The study protocol conformed to the ethical guidelines of the 1975 Declaration of Helsinki. This study was approved by the local Ethic Committee of Sant’Orsola-Malpighi Hospital, Bologna (No. 164/2017/O/Oss). All patients provided written informed consent.

### Statistical analysis

The primary endpoint was PFS, with OS also assessed as secondary endpoint. PFS was calculated from the date of the start of regorafenib to the radiological and/or clinical evidence of disease progression; OS was calculated from the date of the start of regorafenib treatment to the date of death from any cause or last follow-up visit. Individuals alive or with an unknown vital status were censored at the date of their last follow-up or at the cut-off date of 30 September 2020.

In descriptive statistics, continuous variables were reported as the median and 25th-95th percentile, whereas categorical variables were reported as absolute and percentage frequencies.

The Kaplan–Meier estimates were used to calculate survival probability and the log-rank test to make comparisons between curves. The median follow-up time was calculated using the reverse Kaplan–Meier method. The prognostic performance of each covariate on PFS and OS was first evaluated by means of the Cox proportional hazard univariate model, selecting those variables with a *P* value <0.05 for multivariate analysis. For all tests, a two-sided *P* value <0.05 was considered to be statistically significant, with a CI at 95% (95% CI).

The statistical analyses were performed using the SPSS software (version 26; SPSS Inc., Chicago, IL, USA).

## Results

### Patient population features

Overall, 152 patients with GIST fulfilling the inclusion criteria were included in the analysis. The median age was 58 years (range 19-78 years); 82 were male (54%) and 70 (46%) were females. Patients’ characteristics are listed in Table 1.

**Table 1:** Baseline characteristics of patients (*N* = 152)

Patients	Value
Sex, *n* (%)	
Male	82 (54)
Female	70 (46)
Age (years), median (range)	58 (19-78)
Primary tumor site, *n* (%)	
Stomach	48 (31.6)
Duodenum	12 (7.9)
Jejunum	17 (11.2)
Ileum	57 (37.5)
Colon	2 (1.3)
Rectum	6 (3.9)
Extragastrointestinal	10 (6.6)
Risk, *n* (%)	
Low	5 (3.3)
Intermediate	21 (13.8)
High	104 (68.4)
Unknown	22 (14.5)
Mutational status, *n* (%)	
KIT exon 9	31 (20.4)
KIT exon 11	97 (63.8)
KIT exon 17	6 (3.9)
Disease status at diagnosis, *n* (%)	
Localized	78 (51.3)
Metastatic	74 (48.6)
Adjuvant treatment, *n* (%)	
Yes	51 (33.5)
No	101 (66.4)
Site of metastases, *n* (%)	
Liver	42 (27.6)
Peritoneum	31 (20.4)
Liver and peritoneum	54 (35.5)
Other	25 (16.4)
Number of prior lines of therapy, median (range)	3 (2-5)

### Data on regorafenib schedule

A total of 114 (75%) patients were initially treated with regorafenib at the standard dose (160 mg daily, 3-weeks-on, 1-week-off schedule), while 38 (25%) patients had received personalized treatment upfront for clinical reasons [Eastern Cooperative Oncology Group (ECOG) performance status, age, comorbidities, toxicity to previous treatments]. Among the 114 patients initially treated with the standard schedule, 65 (57%) received at least one treatment adjustment due to regorafenib-related AEs. Conversely, only 49 patients (43%) continued the standard dose and schedule until progressive disease. Thus, according to the design of the study, the patients were split into two groups: patients treated with standard dose (*n* = 49) and patients with GIST who received personalized schedules (*n* = 103).

The median time between the start of treatment and the first dose adjustment was 2.3 months (range 0.6-19.7 months).

**Figure 1:**
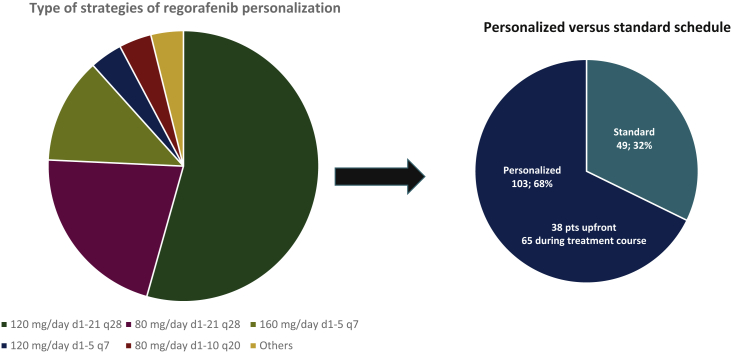
Type of strategies of regorafenib personalization observed in the study population. Pts, patients.

Among the 103 patients for whom treatment was personalized, the following strategies were observed (Table 2 and Figure 1): 120 mg/day d1-21 q28 (*n* = 56; 54.4%); 80 mg/day d1-21 q28 (*n* = 22; 21.4%); 160 mg/day d1-5 q7 (*n* = 13; 12.6%); 120 mg/day d1-5 q7 (*n* = 4; 3.8%); 80 mg/day d1-10 q20 (*n* = 4; 3.8%); others (*n* = 4; 3.8%).

**Table 2:** Type of regorafenib personalization strategies adopted in clinical practice (*N* = 103)

Type of schedule	Value
120 mg/day d1-21 q28	56
80 mg/day d1-21 q28	22
160 mg/day d1-5 q7	13
120 mg/day d1-5 q7	4
80 mg/day d1-10 q20	4
Others	4
Number of dose adjustments	
1	58
2	33
3	7
4	1

In all patients who received a treatment personalization, a reduction or resolution of AEs with an overall improvement in quality of life (QoL) was self-reported.

### Survival outcomes and prognostic factors

**Figure 2:**
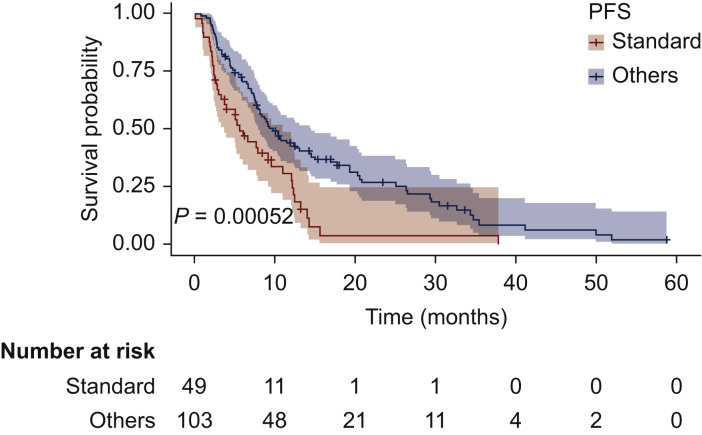
Progression-free survival (PFS) of patients with gastrointestinal stromal tumor (GIST) receiving standard schedule of regorafenib (yellow) or personalized treatment (blue).

**Figure 3:**
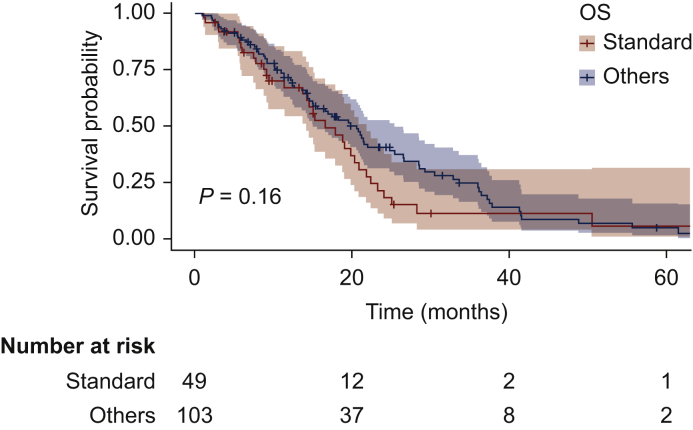
Overall survival (OS) of patients with gastrointestinal stromal tumor receiving standard schedule of regorafenib (yellow) or personalized treatment (blue).

At a median follow-up of 36.5 months, median PFS was 5.6 months (95% CI 3.73-11.0 months) versus 9.7 months (95% CI 7.9-14.5 months) in the standard-dose and the personalized schedule groups, respectively (HR 0.51; 95% CI 0.34-0.75; *P* = 0.00052; Figure 2). Median OS was 16.6 months (95% CI 14.1-21.8 months) versus 20.5 months (95% CI 15.0-25.4 months), respectively (HR 0.75; 95% CI 0.49-1.22; *P* = 0.16) (Figure 3).

At multivariate analysis, PFS was significantly correlated with personalized schedules (HR 0.41; 95% CI 0.24-0.7; *P* = 0.001) (Supplementary Appendix S1, available at https://doi.org/10.1016/j.esmoop.2021.100222).

## Discussion

Even though regorafenib represents an effective treatment option for patients with metastatic GIST, its recommended schedule (160 mg daily, 3-weeks-on, 1-week-off) has the major drawback of unsatisfactory patient adherence due to relevant, sometimes hardly bearable, treatment-related AEs.^19^, ^20^, ^21^, ^22^, ^23^ In order to tackle this issue, physicians usually adopt various dosing and schedules of regorafenib, in order to optimize treatment and avoid early discontinuation.^16^^,^^18^^,^^20^ However, to the best of our knowledge, no data on the impact of regorafenib-personalized schedules on therapeutic outcomes in patients with advanced GIST have been published. Moreover, regorafenib treatment management seems still extremely heterogeneous and mainly based on each oncologist’s own clinical experience. The results of our multicenter, retrospective, real-world study confirmed that regorafenib-personalized schedules are commonly adopted in everyday clinical practice of high-volume GIST expert centers. Indeed, only one-third of patients received standard schedule of regorafenib without treatment modifications.

This report showed for the first time that regorafenib treatment personalization may correlate with statistically significant improvement of therapeutic outcomes. Indeed, median PFS of patients treated with personalized schedules since the beginning or following treatment adjustment due to intolerance was 9.7 months, in comparison to 5.6 months in patients receiving standard schedule, which is in line with data of the landmark GRID trial. Interestingly, the gain in median PFS was found regardless of the number of previous therapies, highlighting the relevance of treatment personalization during all disease course.

Moreover, a trend for survival benefit in patients treated with personalized treatment (20.5 months versus 16.6 months) has been observed. However, the lack of a statistically significant difference could be explained by the small sample size analyzed as well as the expected impact on survival of all treatments received after regorafenib, which have progressively increased during the years.

The time interval between the beginning of regorafenib treatment and the first dose modification is another aspect to consider. According to our results, the median time was slightly higher than 2 months (2.3 months), confirming that most regorafenib-related AEs occur early within the first weeks of starting treatment, regardless of primary malignancy. For example, in the REBECCA trial evaluating regorafenib in patients with colorectal cancer in a real-life setting—the median time to first dose modification was 0.7 months, and these data have been confirmed also in other clinical trials assessing the role of regorafenib in different malignancies, including GIST.^21^, ^22^, ^23^ Thus a prompt personalization of treatment as well as a close monitoring during the first cycles of regorafenib could help avoid early treatment discontinuation due to AEs.

The main limitations of this study are the relatively small sample size and the retrospective design. Moreover, the wide heterogeneity of all personalized strategies does not allow to define which one has the best safety profile-to-disease control ratio in patients with GIST. On the basis of these considerations, our results should be interpreted with caution given these limitations and additional good-quality randomized clinical trials with larger cohorts of patients will be needed to confirm our findings. However, this multicenter experience, the largest so far on this challenging topic and on a rare tumor, provides a benchmark for future trials assessing personalization strategies in this setting. Of note, the actual efficacy of personalized schedules in metastatic GIST is virtually unknown outside of clinical trials.

In addition, these findings could be useful in daily clinical practice, helping physicians to maximize the use of regorafenib in patients with GIST, without early discontinuation for unmanageable toxicity. Certainly, there is a high unmet need to optimize the dosing schedule of regorafenib in patients with metastatic GIST, in order to allow maintenance of antitumor efficacy, without neglecting the tolerability profile and the associated quality of life—an extremely important issue especially in the metastatic setting, where cure is no longer possible and maintaining control of symptoms represents a key need.

### Conclusions

This retrospective multicenter study shows that regorafenib-personalized schedules are commonly adopted in daily clinical practice and correlate with statistically significant improvement of therapeutic outcomes. This report highlights the relevance of patient-tailored approaches that could be applied to other metastatic solid tumors treated with this drug, for which the maximization of treatment and patient quality of life are key goals.
