# Supplementary material for: Standard versus personalized schedule of regorafenib in metastatic gastrointestinal stromal tumors: a retrospective, multicenter, real-world study
Source: ESMO Open. 2021 Aug 2;6(4):100222. doi: 10.1016/j.esmoop.2021.100222 (PMC8350191; doi:10.1016/j.esmoop.2021.100222)
Supplement: Supplementary Appendix [file mmc1.docx]

**Supplementary Table 1. Multivariate Analysis for Factors affecting PFS. Significant factors are depicted in bold.**

|  | **Univariate** | **Multivariate** | |
| --- | --- | --- | --- |
| **Variable** | **p Value** | **HR (95% CI)** | **p Value** |
| Personalized schedules | **0.02** | 0.41 (0.24-0.7) | **0.001** |
| Age | **0.03** | 1.02 (1.00-1.04) | 0.071 |
| Number of previous treatments | **0.04** | 0.89 (0.66-1.17) | 0.38 |
| Adjuvant therapy (yes) | **0.03** | 1.01(0.61-1.67) | 0.973 |
| Risk Class: High | **0.04** | 0.59 (0.2-1.77) | 0.35 |
| Risk Class: Intermediate | **0.02** | 0.43 (0.12-1.58) | 0.21 |
| Primary tumor site: Stomach | **0.03** | 1.38 (0.46-4.11) | 0.56 |
| Primary tumor site: Duodenum | **0.04** | 2.89 (0.79-10.51) | 0.11 |
| Primary tumor site: Jejunum | **0.03** | 1.22 (0.34-4.35) | 0.77 |

**Supplementary Table 2. Multivariate Analysis for Factors affecting OS.**

|  | **Univariate** | **Multivariate** | |
| --- | --- | --- | --- |
| **Variable** | **p Value** | **HR (95% CI)** | **p Value** |
| Personalized schedules | **0.03** | 0.75 (0.44-1.29) | 0.3 |
| Age | **0.04** | 1.01 (0.99-1.03) | 0.27 |
| Number of previous treatments | **0.04** | 1.03 (0.76-1.41) | 0.83 |
| Adjuvant therapy (yes) | **0.03** | 1.27 (0.72-2.22) | 0.41 |
| Risk Class: High | **0.04** | 1.73 (0.58-5.2) | 0.32 |
| Risk Class: Intermediate | **0.03** | 1.6 (0.48-5.72) | 0.47 |
| Primary tumor site: Stomach | **0.04** | 1.76 (0.47-6.53) | 0.4 |
| Primary tumor site: Duodenum | **0.04** | 1.67 (0.39-7.71) | 0.49 |
| Primary tumor site: Jejunum | **0.04** | 1.74 (0.37-5.33) | 0.47 |
